# Is traditional Chinese medicine service accessibility associated with health inequality? Evidence from the distribution of health capital among rural residents in China

**DOI:** 10.1186/s12939-026-02942-8

**Published:** 2026-07-07

**Authors:** Na Xiong, Yingxin Lin, Haitao Cui

**Affiliations:** School of Management, Guangxi Minzu University, Nanning, Guangxi China

**Keywords:** Traditional Chinese medicine accessibility, Health capital, Health inequality, Rural residents, Healthcare resource allocation

## Abstract

**Background:**

Health inequality remains a persistent challenge in rural areas, where limited healthcare accessibility is often associated with lower levels of health capital. In China, traditional Chinese medicine (TCM) service accessibility constitutes an important component of the rural healthcare system. However, whether and to what extent TCM service accessibility is associated with health capital and health inequality remains insufficiently understood. This study investigates whether, and through which mechanisms, TCM service accessibility is associated with health capital among rural residents and whether it is associated with lower health inequality.

**Methods:**

This study uses micro-level survey data from 12860 rural residents across six provinces in China, combined with county-level healthcare resource statistics. The empirical strategy includes multivariate regression, instrumental variable estimation, mediation analysis, heterogeneity analysis, and inequality decomposition. Health capital is measured using a composite index constructed from four standardized dimensions: self-rated health, activities of daily living, chronic disease status, and objective physiological indicators (including blood pressure and fasting blood glucose). These indicators are aggregated using principal component analysis (PCA), with the first principal component used as the health capital index. TCM service accessibility is operationalized along three dimensions: supply availability, geographical accessibility, and individual utilization.

**Results:**

The results show that greater TCM service accessibility is significantly associated with higher levels of health capital among rural residents. Specifically, a 0.1-unit increase in TCM service accessibility corresponds to an increase of approximately 0.021 units in the health capital index. The estimated effects exhibit significant heterogeneity, with larger magnitudes among individuals with poorer baseline health and lower income levels, and in settings with higher levels of social support. From an equity perspective, TCM service accessibility shows an equalizing association with the distribution of health capital, with a contribution share of −0.10 and an estimated contribution corresponding to approximately 0.030 of the health capital Gini coefficient. This represents the largest equalizing association among all examined factors, including income, education, and Western medical resources. Quantile regression results further indicate that the estimated effect at the 10th percentile of the health capital distribution is four times larger than that at the 90th percentile, suggesting larger estimated associations for disadvantaged groups. Mediation analysis suggests that healthcare utilization and health behavior are key potential pathways linking TCM service accessibility to health capital, with the total mediating effect accounting for 47.6% of the overall association.

**Conclusions:**

The findings suggest that TCM service accessibility is associated with higher health capital and a more equitable distribution of health capital among rural residents. These findings highlight the equity-enhancing potential of traditional medicine within pluralistic healthcare systems and provide important policy implications for addressing persistent health disparities in resource-constrained rural settings.

## Introduction

Health inequality is a persistent structural challenge for health systems worldwide. Rural residents are often at a relative disadvantage in terms of healthcare accessibility, chronic disease burden, and health outcomes. Existing studies show that in many resource-constrained settings, rural populations are more likely to face multiple constraints, including insufficient healthcare supply, limited accessibility, and high financial burdens, resulting in consistently poorer health outcomes compared with urban populations [1]. In China, since the implementation of the “Healthy China 2030” strategy, per capita health expenditure in rural areas has continued to increase [2]. However, disparities in healthcare resource allocation between urban and rural areas remain substantial [3]. Therefore, identifying healthcare resources that are both accessible and cost-effective in rural areas, and examining how they relate to health capital and the distribution of health, is of important practical significance.

Health capital refers to a durable stock that individuals possess, which depreciates with age and can be maintained or enhanced through investment. It affects not only individual quality of life but also labor capacity, household welfare accumulation, and the ability to cope with health risks, and is a core component of human capital [4]. For rural residents, insufficient health capital is often associated with a higher burden of chronic diseases, limited healthcare accessibility, and an increased risk of poverty due to illness [5]. Within China’s rural healthcare system, traditional Chinese medicine (TCM) has long been an important component of primary healthcare services. Early World Health Organization (WHO) reports on the role of traditional medicine in primary healthcare in China, as well as the WHO Global Report on Traditional and Complementary Medicine (2019), highlight the sustained role of traditional medicine, including TCM, in primary care, chronic disease management, and meeting the health needs of aging populations [6, 7]. According to the Statistical Communiqué on the Development of China’s Health and Health Services (2023), 99.6% of township health centers and 82.9% of village clinics provide TCM services, indicating that TCM service accessibility has achieved a high level of coverage within rural primary healthcare institutions [8]. However, existing studies on TCM have mainly focused on clinical effectiveness, utilization preferences, or urban–rural resource differences [7, 9]. There is still a lack of systematic micro-level empirical evidence on whether TCM service accessibility is associated with health capital among rural residents, and whether this association is related to lower health inequality within rural areas.

Specifically, two key research gaps remain in the literature. First, although a large body of research has examined the relationship between Western medical services and health capital [10, 11], the role of traditional medicine in shaping health capital has received limited attention, and the potential pathways linking TCM service accessibility to health capital among rural residents remain unclear. Second, existing studies on healthcare accessibility and health equity have largely focused on urban–rural disparities [12, 13], with relatively little attention given to health inequality within rural populations. It remains unclear whether TCM, as a complementary healthcare resource, is associated with a more equitable distribution of health capital among different groups within rural areas. To address these gaps, this study focuses on rural China and examines two central research questions: (1) Is TCM service accessibility associated with health capital among rural residents, and through what mechanisms? (2) Does this association vary across population groups, and is TCM service accessibility associated with lower health inequality within rural areas?

It is important to distinguish between urban–rural disparities and within-rural inequality, as they represent different levels of analysis. Existing research on healthcare equity has mainly focused on average differences between urban and rural areas, such as disparities in resource allocation, healthcare utilization, and health outcomes [14, 15]. In contrast, within-rural inequality refers to differences in the distribution of health capital among rural populations with different income levels, initial health endowments, and resource accessibility. Studies in western rural China have shown that significant income-related health inequality exists within rural populations, with income and education identified as key determinants [16]. This implies that even when overall rural healthcare resources improve, disparities may persist if the benefits are disproportionately concentrated among individuals with better initial health or socioeconomic conditions. Therefore, shifting the analytical focus from “whether rural areas are disadvantaged relative to urban areas” to “which groups within rural areas benefit more from improvements in healthcare resources” constitutes the core perspective of this study and represents an incremental contribution to the existing literature.

This study contributes to the literature in three main aspects. First, it provides micro-level empirical evidence on the relationship between traditional medical services and health capital in the context of rural China, extending beyond existing studies that mainly focus on modern healthcare services and high-income countries [17, 18]. Second, it shifts the focus from average urban–rural differences to the distribution of health within rural areas, examining whether TCM service accessibility is associated not only with health levels but also with health inequality, thereby extending the distributional perspective in healthcare accessibility research. Third, it integrates health capital theory, healthcare accessibility, and health equity analysis to examine the potential pathways linking TCM service accessibility to health capital, including healthcare utilization and health behavior. While this study is based on the rural China context, its applicability to other developing regions requires further comparative research.

From a practical perspective, this study provides empirical evidence for optimizing the allocation of healthcare resources in rural China. Official statistics indicate that TCM services have achieved high coverage in primary healthcare institutions; however, differences in accessibility may still exist across regions, institutional levels, and population groups [8]. Examining the relationship between TCM service accessibility, health capital, and health inequality can provide evidence that may inform primary healthcare service improvement and health equity policy design.

## Literature review

### Analytical foundations: health capital theory, health inequality, and healthcare accessibility

Health capital theory was first proposed by Grossman (1972). This framework moves beyond the traditional view of health as merely a consumption outcome and conceptualizes health as a capital stock with both consumption and investment attributes. Individuals can maintain and improve health capital through healthcare utilization, investments in health behavior, and time allocation, thereby maximizing intertemporal utility [4]. Subsequent studies have further extended health capital theory by emphasizing its cumulative and dynamic nature over the life course, as well as its long-term effects on education, income, and social participation. Existing evidence suggests that early-life health conditions not only influence health status in adulthood but also generate persistent effects through pathways such as labor capacity, educational attainment, and income opportunities. These effects tend to be more pronounced in resource-constrained developing contexts [10, 17].

In terms of measurement, the literature has developed a three-dimensional framework of objective, subjective, and composite indicators. Objective indicators typically include population-level measures such as mortality, life expectancy, or disability-adjusted life years. While these indicators facilitate macro-level comparisons, they are limited in capturing individual-level heterogeneity in health capital. Subjective indicators, most commonly self-rated health, have been shown to be broadly consistent with objective health and clinical measures, but are inevitably influenced by individual perception, social comparison, and cultural context [19, 20]. Composite indicators aim to integrate multidimensional health information to better capture the complexity of health capital. Existing studies suggest that relying on a single indicator may inadequately capture multidimensional variation in health capital [21]. However, most existing measurement frameworks are developed in general population or urban contexts and may not fully capture the characteristics of rural settings. In rural China, factors such as traditional health beliefs, long-term self-regulation practices, and informal care at the community level may jointly influence health status and its reporting. Therefore, how to more accurately measure health capital in rural contexts remains an issue requiring further research [22].

The theoretical explanations of health inequality draw on multiple disciplines, with differences in resource accessibility occupying a central position. The fundamental cause theory proposed by Link and Phelan (1995) suggests that socioeconomic status continuously shapes health inequality by influencing individuals’ ability to access healthcare resources, health information, and risk-avoidance opportunities [23]. Existing studies show that low-income populations in rural areas often face dual constraints in both geographical and economic dimensions of healthcare accessibility, which are associated with lower health capital and persistent disadvantage [18, 24]. Whitehead (1992) further extends the analytical framework by distinguishing between procedural equity and outcome equity, emphasizing that the evaluation of health inequality should not only consider whether healthcare resources are allocated, but also whether improvements in resources are translated into better health outcomes for disadvantaged groups [25].

From this perspective, health capital theory, health inequality theory, and healthcare accessibility research are closely interconnected. Health capital theory explains why health is a key resource requiring sustained investment; health inequality theory explains why this resource is unevenly distributed across social groups; and healthcare accessibility provides the key mechanism linking the two. Whether healthcare resources can be effectively accessed and utilized is closely related to both health capital levels and the extent to which health capital is equitably distributed. However, existing research on this analytical framework is largely based on modern biomedical services. There remains a lack of systematic integration regarding the role of traditional medicine, particularly in rural primary healthcare settings, in simultaneously shaping health capital and its distribution.

### Healthcare accessibility and health outcomes

Healthcare accessibility is an important factor associated with health outcomes and health capital levels. Its mechanisms are mainly reflected in reducing the depreciation of health capital, improving the efficiency of disease intervention, and mitigating the long-term losses caused by health shocks. From the perspective of geographical accessibility, spatial studies have identified a significant distance-decay effect. Based on survey data from rural areas in the United States, Pathman et al. (2006) show that insufficient healthcare supply significantly increases travel time and transportation costs for residents seeking care [26]. Arcury et al. (2005) further find that geographical factors such as distance to usual sources of care are associated with routine check-ups and chronic disease-related healthcare utilization among rural residents, indicating that spatial accessibility is an important constraint on healthcare utilization in rural areas [27]. Using large-scale county-level data in China, Yin et al. (2018) show that the spatial accessibility of healthcare facilities varies significantly across regions, with poorer accessibility in western areas characterized by complex terrain. Analyses using geographically weighted regression (GWR) suggest that both natural and socioeconomic factors, such as terrain slope and population density, exhibit spatial heterogeneity in their effects on healthcare accessibility, indicating that geographical conditions play a moderating role in shaping healthcare accessibility in rural areas [28].

Economic accessibility is closely related to health capital levels through price mechanisms, particularly in the context of healthcare financing reforms. Existing studies show that high out-of-pocket medical expenses significantly increase the risk of households falling into or remaining in poverty, and the share of healthcare expenditure in income has important implications for equity in health services and the distribution of social welfare, highlighting the importance of reducing financial burden in improving health inequality [29]. Studies on healthcare system reforms in China indicate that reducing out-of-pocket payments has, to some extent, improved the health status of rural residents and reduced their financial burden [30, 31]. Related research also suggests that TCM services, as an important component of the rural healthcare system, play a positive role in improving healthcare accessibility and meeting basic health needs in rural areas, providing empirical evidence for evaluating the economic and social value of traditional medicine [32, 33].

However, existing research on the relationship between healthcare accessibility and health outcomes remains largely centered on the modern biomedical system. Whether focusing on geographical distance, physician density, bed allocation, or health insurance coverage and financial burden, most analyses are based on formal healthcare systems dominated by Western medicine. In contrast, traditional medicine occupies a relatively marginal position in studies of healthcare accessibility, often being examined mainly in terms of cultural preference, complementary treatment, or clinical effectiveness, rather than being incorporated into analytical frameworks of health inequality and health capital. This suggests that while existing studies have provided substantial evidence on why healthcare accessibility in modern medical systems matters, they have not yet fully addressed whether and how TCM service accessibility plays a similar role.

### The role of TCM in the healthcare system: potential value and ongoing debates

TCM has developed a comparative advantage in China’s rural healthcare system due to its characteristics of simplicity, convenience, affordability, and effectiveness. According to official statistics, TCM resources have achieved large-scale coverage. By 2023, 99.6% of township health centers and 82.9% of village clinics provided TCM services. Based on these figures, it is estimated that approximately 516000 rural healthcare institutions provide TCM services, accounting for about 83.8% of all rural healthcare institutions (approximately 615800), indicating that TCM service accessibility has a broad and well-established foundation in rural primary healthcare systems [8]. At the same time, existing studies show that the distribution of TCM resources in China exhibits significant regional disparities. The degree of equality in allocation based on geographical area is substantially lower than that based on population. Related studies consistently find that the Gini coefficient exceeds the commonly used inequality threshold of 0.4, and further estimates suggest that the Gini coefficient of TCM resources based on geographical area typically ranges between 0.62 and 0.65, indicating pronounced spatial inequality [34].

With regard to the role of TCM in the healthcare system, the literature has proposed several potential mechanisms. First, TCM services are generally characterized by relatively low treatment costs, high accessibility at the primary level, and a certain degree of cultural acceptability. These features may provide advantages in chronic disease management, elderly care, and primary intervention for common conditions. Some pharmacoeconomic studies suggest that TCM demonstrates cost-effectiveness advantages in chronic disease management. For example, Xu et al. (2020) show that combined treatment using traditional Chinese herbal medicine and conventional Western medicine for hypertension is more cost-effective than using Western medicine alone, with such advantages being particularly pronounced in resource-constrained or low-income populations [35]. Second, TCM emphasizes prevention, regulation, and long-term care, which gives it potential value in maintaining health capital and shaping health behavior.

However, it should be noted that the evidence on the benefits of TCM is not unidirectional, and conclusions should be interpreted with caution. First, the quality of evidence on effectiveness and cost-effectiveness varies across diseases, intervention types, and research designs, and positive findings in specific disease areas cannot be generalized [36]. Second, there may be substantial heterogeneity in provider quality in the delivery of TCM services at the primary level, with differences across regions, institutions, and practitioners in service capacity, standardization, and continuity of care [37]. Third, in certain contexts, particularly for conditions requiring timely standardized treatment, reliance on TCM as a substitute for, or delay in accessing, more effective biomedical treatment may lead to adverse health outcomes [38, 39]. Fourth, in many disease contexts, TCM is more commonly positioned as a complementary or integrative intervention rather than a substitute for standard biomedical treatment, and its effectiveness depends on how well it is integrated with primary biomedical services [40, 41]. Therefore, it is not sufficient to assume that TCM service accessibility is inherently beneficial; its effects need to be empirically examined within specific institutional and local contexts.

Existing research on TCM and health is subject to limitations in both analytical perspective and methodology. From the perspective of research focus, most studies emphasize clinical effectiveness, such as the mechanisms of herbal medicine or the neuroregulatory effects of acupuncture, while research from a health economics perspective remains limited. Notably, the application of resource deprivation theory is largely absent. From a methodological perspective, many studies rely on case analysis or small-sample surveys, lacking large-scale causal identification strategies. Although some studies have used data from the China Family Panel Studies to examine cross-sectional relationships between healthcare utilization and health status, these studies typically do not focus on the mechanisms through which TCM service utilization affects health outcomes, and they face challenges in addressing endogeneity issues, such as reverse causality and differences between geographical accessibility and actual utilization [42].

Overall, three gaps remain in the existing literature. First, most studies rely on cross-sectional designs and provide limited evidence on potential endogeneity. Second, the pathways through which TCM service accessibility may be associated with health capital remain insufficiently examined. Third, evidence on whether TCM accessibility is associated with within-rural health inequality remains limited. These gaps motivate the present study.

## Theoretical framework and research hypotheses

### TCM service accessibility and health capital

According to the theory of health human capital, health capital is a durable stock with both investment and consumption attributes. Its dynamic accumulation follows a stock–flow model: the current level of health capital equals the previous stock plus current health investment minus the depreciation of health capital.

**Fig. 1:**
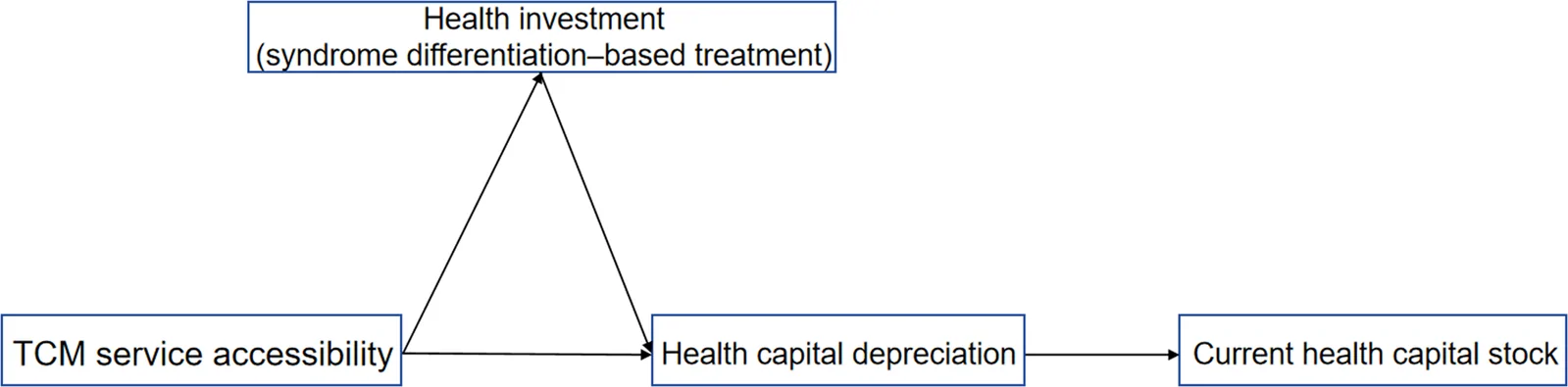
Stock–flow model of health capital depreciation and investment

TCM service accessibility may influence health capital through two potential pathways. First, it may be linked to the depreciation process of health capital through intervention-related mechanisms. Second, it may be associated with health investment through behavioral responses. Through these pathways, TCM service accessibility may be related to higher levels of health capital. The theoretical mechanism is illustrated in Fig. 1 (stock–flow model of health capital depreciation and investment).

The direct intervention pathway concerns the potential relationship between TCM service accessibility and the maintenance of health capital. TCM emphasizes prevention, regulation, and individualized care. Existing studies suggest that preventive TCM interventions may contribute to chronic disease prevention and health maintenance [43]. In addition, TCM-based approaches have been applied in the management of common chronic conditions, including hypertension, diabetes, and chronic respiratory diseases [44]. Systematic reviews indicate that some TCM interventions may be associated with improvements in blood pressure, blood glucose control, and chronic symptom management [45, 46]. As a distinctive feature of TCM, syndrome differentiation and individualized treatment have also been discussed as potential contributors to health management and recovery processes [47, 48]. From a health capital perspective, these mechanisms may be associated with slower health depreciation and better maintenance of health status [4].

**Fig. 2:**
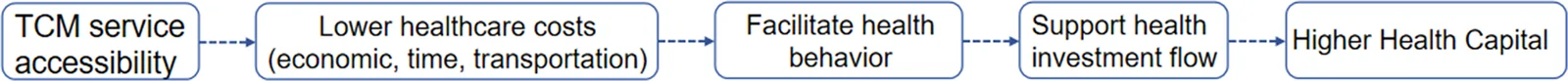
Mechanisms linking TCM service accessibility to health capital

The indirect pathway, as shown in Fig. 2, may reflect the association between the accessibility advantages of TCM services, healthcare utilization, health behavior, and health capital among rural residents. From a cost perspective, compared with some biomedical examinations and treatments, TCM services—such as herbal decoctions, acupuncture, and massage—generally involve lower per-visit costs. In addition, commonly used herbal medicines at the primary level can often be locally sourced, which may lower the economic cost of healthcare for rural residents and may be associated with less foregone care due to financial constraints. Existing studies and official statistics indicate that TCM services are widely embedded in the rural primary healthcare system and have achieved high coverage in village clinics and township health centers [49]. The local availability of primary healthcare institutions significantly shortens travel distance, thereby reducing time and transportation costs and improving healthcare accessibility and the convenience of healthcare utilization [50, 51]. From the perspective of health behavior and health capital theory, lower time and financial costs of healthcare may be associated with more sustained health behavior and higher health capital through investments in prevention, treatment, and rehabilitation [52]. Previous studies suggest that improved healthcare accessibility contributes to more continuous health management behavior [53]. In rural China, TCM services are widely used across multiple stages, including disease prevention, treatment, and rehabilitation, and may therefore be relevant to sustained health behavior among rural residents [50].

Based on the stock–flow model of health capital, we propose the following core hypothesis:

#### H1:

Greater TCM service accessibility is positively associated with the health capital level of rural residents.

### Heterogeneity and the moderating role of social support

Differences in initial health endowments and economic resources among rural residents may be associated with variation in both the demand for and responsiveness to TCM service accessibility, resulting in heterogeneous associations. On the one hand, a stronger association among disadvantaged groups may exist among low-income populations. Rural residents with lower income face stronger financial barriers to healthcare and may forgo treatment due to the high cost of biomedical services, which may be associated with lower health capital levels. Medical expenditure is therefore a key constraint on healthcare utilization among low-income groups, and lower treatment costs may be associated with less forgone care due to financial constraints [54]. This mechanism is consistent with the established finding in health economics that the price elasticity of healthcare demand is higher among low-income populations [55]. On the other hand, a “marginal improvement” effect exists among individuals with poorer initial health. Groups with lower baseline health status, such as those with chronic conditions or weaker physical conditions, have greater potential for health improvement. Compared with healthier individuals, their lower initial health capital implies that relatively small interventions may generate larger marginal effects. The individualized approach of TCM, such as tailored treatments for specific conditions, may allow for targeted interventions that alleviate symptoms and improve physical functioning, resulting in stronger marginal effects of TCM service accessibility for these groups [56].

Based on the literature on social support, family and social support are associated with higher treatment adherence. In community healthcare settings, trust in TCM preventive and health management services is related to the availability of local service provision, and social relationships and networks are positively associated with beliefs in and use of TCM [57–59]. At the community level, activities such as health lectures on TCM and public consultation services can help improve understanding and increase trust in TCM services, thereby encouraging more active healthcare utilization. At the household level, basic knowledge of TCM among family members may facilitate daily health practices and mutual support, strengthening the continuity of health behavior and enhancing the effects of TCM service accessibility [60]. The reinforcing effect of family and social support on healthcare utilization has been documented in previous studies on health behavior [57, 61].

Based on the above analysis, the positive association between TCM service accessibility and health capital may vary by individual characteristics and social support. We propose the following hypotheses:

#### H2:

The association between TCM service accessibility and health capital level varies across groups and social support contexts.

#### H2a:

The lower the initial health status, the stronger the positive association between TCM service accessibility and health capital level.

#### H2b:

The lower the income level, the stronger the positive association between TCM service accessibility and health capital level.

#### H2c:

The higher the level of social support, the stronger the positive association between TCM service accessibility and health capital level.

### TCM service accessibility and health inequality

The relationship between TCM service accessibility and the distribution of health capital concerns not only whether higher levels of health capital are observed among individuals, but also whether health capital is distributed more evenly across groups [25]. TCM service accessibility may be associated with disparities in health capital through two channels: procedural equity (spatial allocation) and outcome equity (differential marginal effects).

From the perspective of spatial mismatch theory, greater TCM service accessibility in rural areas may help alleviate spatial misallocation of healthcare resources and may be associated with more equitable healthcare accessibility for disadvantaged groups. At present, there are significant spatial disparities in TCM services across rural areas, with higher service density, better-qualified practitioners, and higher-quality herbal resources in eastern rural regions compared with western regions, and in peri-urban rural areas compared with remote areas. Measures to improve TCM service accessibility, such as increasing the number of TCM practitioners in western rural areas, supplying herbal medicines to remote village clinics, and developing internet-based and telemedicine TCM services, may help alleviate these spatial imbalances. These improvements may allow rural residents in low-income and remote areas to access TCM services under more comparable conditions to those in higher-income or peri-urban areas, thereby providing a potential procedural basis for a more equitable distribution of health capital [62].

From the perspective of health equity theory, the association between TCM service accessibility and health capital may be stronger among disadvantaged groups, suggesting a potential equalizing pattern [25]. For individuals with lower income and poorer baseline health, this association may appear stronger, possibly because these groups face greater chronic disease burdens and health-related risks. This pattern, in which the association appears stronger among disadvantaged groups, suggests a potential equalizing role in the distribution of health capital across groups [63].

Based on the above analysis, we propose the following equity-related hypothesis:

#### H3:

Greater TCM service accessibility is associated with lower health inequality among rural residents.

## Study design

### Study area

This study focuses on rural areas in China. Based on considerations of regional representativeness and the need to examine heterogeneity, a multi-stage stratified sampling approach is used to select rural counties from six provinces: Shandong and Jiangsu in eastern China, Henan and Hubei in central China, and Sichuan and Gansu in western China. In terms of regional characteristics, the selected provinces cover diverse geographical conditions, including plains, hilly areas, and mountainous regions. In 2023, the proportion of the rural population in all selected provinces remained above 40%, and the development of TCM services shows clear regional variation. The coverage of TCM services in rural areas is approximately 82% in eastern regions, 70% in central regions, and 58% in western regions. This gradient distribution reflects regional differences in TCM service accessibility and health capital levels in rural China. In addition, differences across the six provinces in terms of traditional medical culture and the development of primary healthcare systems provide a suitable setting for examining regional heterogeneity in the effects of TCM service accessibility, consistent with the logic of cross-regional comparative analysis.

### Data sources and processing

To support multi-level analysis, this study adopts a data-matching strategy that combines individual-level survey data with county-level healthcare resource data. Individual-level data are drawn from the 2022 wave of the China Family Panel Studies (CFPS), conducted by the Institute of Social Science Survey at Peking University. The CFPS employs a multi-stage probability sampling design covering 25 provinces, autonomous regions, and municipalities in China, and includes information on health status, healthcare utilization, household economic conditions, and community environments. Because this study is based on cross-sectional data, it cannot directly capture the dynamic process of health capital accumulation. Future research may incorporate panel data from multiple CFPS waves to examine dynamic effects. After data cleaning, a total of 12860 valid observations of rural residents are obtained.

County-level variables on TCM and Western medical resources are obtained from official health statistical sources, such as county-level health statistics yearbooks or comparable databases. To avoid inconsistencies due to time mismatch, county-level indicators from the same year as CFPS or from the preceding year are matched to the individual-level data. The county-level dataset covers 186 rural counties across the six selected provinces, with no missing values.

Individual-level data and county-level data are merged using county administrative codes, resulting in a two-level dataset with an “individual–county” structure. As the key explanatory variable is measured at the county level, all regression models use cluster-robust standard errors at the county level to account for within-county correlation. In addition, regional fixed effects are included through county dummy variables to control for unobserved factors at the regional level that do not vary across individuals.

The World Health Organization database on traditional medicine, as well as health statistics from India and South Africa, are used only as references for defining objective physiological indicator thresholds and for providing background in qualitative comparison. Specifically, WHO standards are used to define normal ranges for indicators such as systolic blood pressure and fasting blood glucose. The international data are not included in the core econometric models. To avoid ambiguity regarding the scope of the study, these data are not used in the empirical analysis and are only briefly referenced in the discussion as contextual background.

### Instrumental variable data

To address potential endogeneity between TCM service accessibility and health capital, such as the possibility that individuals with better health are more likely to use TCM services, this study constructs two instrumental variables based on historical persistence and exogeneity, using publicly available historical records and official databases.

The first instrumental variable is the number of historically recognized TCM practitioners at the county level before 1949. These data are collected from publicly available historical sources, including the China Yearbook of Traditional Chinese Medicine and county-level gazetteers. The China Yearbook of Traditional Chinese Medicine covers the period from 1989 to 2023 and is available through the official website of China Traditional Chinese Medicine Press. County gazetteers, including local health and cultural records, are accessed through the Chinese Local Gazetteers Database of the National Library of China. The sample includes 186 counties, and the variable captures the number of well-documented TCM practitioners before 1949, including hereditary practitioners, descendants of imperial physicians, and locally recognized TCM doctors.

To support the exogeneity of this variable, the following tests and clarifications are provided. First, the correlations between this variable and historical human capital (proxied by the number of private schools before 1949) and cultural attitudes toward health (proxied by the proportion of residents reporting regular health check-ups) are 0.12 and 0.09, respectively, both statistically insignificant. This provides some reassurance that the instrument is not strongly correlated with these measured alternative channels, although unobserved pathways cannot be fully ruled out. Second, after controlling for historical socioeconomic development at the county level (proxied by the total industrial and agricultural output index in 1952), the first-stage coefficient of the instrumental variable remains significantly positive (coefficient = 0.29, *p* < 0.01), and the estimated effect of TCM service accessibility on health capital remains robust. This provides supportive evidence that the instrument is not strongly correlated with this measured historical socioeconomic factor, although the exclusion restriction remains an identifying assumption.

The second instrumental variable is the number of TCM-related intangible cultural heritage (ICH) items at the county level, obtained from the official database of the Ministry of Culture and Tourism of China. This database provides information on nationally and provincially recognized intangible cultural heritage items, including practices such as acupuncture and traditional herbal processing techniques. The number of such items in each county is used as an alternative instrumental variable for robustness checks.

It should be noted that the selected TCM ICH items are all historically inherited practices, with recognition criteria emphasizing the continuity of traditional techniques formed from the Ming and Qing dynasties to the Republican period, and are not directly related to contemporary regional policies or cultural investment. For example, among the first batch of nationally recognized TCM ICH items in 2006, more than 80% originated from traditions with over a century of history and were not newly created through modern policy initiatives. In addition, the correlations between this variable and current regional economic development, measured by gross domestic product (GDP) per capita, and healthcare system investment are 0.07 and 0.10, respectively, both statistically insignificant, providing additional support against this specific source of concern. For the specification with two instrumental variables, the Sargan overidentification test results are reported in Table 3 (χ^2^ = 1.72, *p* = 0.186), failing to reject the null hypothesis that all instruments are exogenous, which further supports the validity of the exclusion restriction.

Although these tests provide supportive evidence for instrument validity, the exclusion restriction cannot be directly verified. Historical TCM traditions may be associated with health capital through alternative pathways, including local health culture, socioeconomic conditions, public service provision, and preventive health preferences. While we control for a broad set of county-level and individual-level factors and conduct additional validity checks, the exclusion restriction should be interpreted as a plausible assumption rather than a testable fact.

### Variable definition and measurement

Based on the theoretical frameworks of health capital theory and social support theory, and consistent with the core analytical logic of this study, we construct a multidimensional variable system including the dependent variable, key explanatory variable, mediating variables, moderating variables, and control variables. The definitions and measurement of each variable are described as follows.

#### Dependent variable: health capital

Health capital, as a comprehensive representation of individual health status, is grounded in the health capital model (Grossman, 1972). In empirical measurement, it is necessary to incorporate both subjective health assessments and objective health outcomes, while capturing functional status and physiological conditions [4, 19]. The inclusion of subjective indicators such as self-assessed health and self-reported chronic conditions may introduce reporting bias, as widely discussed in the literature, due to differences in health perception across population groups [64, 65]. To mitigate this bias, this study adopts three strategies. First, a multidimensional framework combining subjective evaluation and objective indicators is used. Objective measures, including activities of daily living (ADL), systolic blood pressure, and fasting blood glucose, serve to anchor health status and reduce uncertainty associated with purely subjective measures. Second, during the standardization process, region-specific standardization is applied across eastern, central, and western regions to reduce systematic bias arising from differences in health perception. Third, principal component analysis (PCA) is used to extract a common factor, and the consistency of factor loadings across subjective and objective indicators is used to assess measurement stability, thereby avoiding the amplification of bias from any single subjective indicator.

Although objective indicators such as chronic disease status, blood pressure, and blood glucose may be influenced by individual use of TCM, this study addresses potential endogeneity through the instrumental variable approach. In the core regression models, TCM service accessibility is defined as a supply-side and geographical variable. Healthcare utilization and health behavior are treated as mediating variables, distinguishing supply-side accessibility from demand-side behavioral responses and reducing potential simultaneity bias.

Based on existing studies and the availability of data from the 2022 wave of CFPS, the health capital index is constructed from four dimensions as follows.

Self-rated health (SRH) is measured using a five-point scale in CFPS (“How would you rate your current health status?”), coded as 1 = very poor, 2 = poor, 3 = fair, 4 = good, and 5 = very good. The original scores are normalized to a 0–1 scale, with higher values indicating better subjective health. Activities of daily living (ADL) are based on five basic activities in CFPS, including dressing, bathing, eating, toileting, and mobility. A binary coding is used (no difficulty = 1, difficulty = 0). The proportion of activities with difficulty is calculated and then reverse standardized to a 0–1 scale, with higher values indicating better functional status. Chronic disease status (CDS) is defined based on major chronic conditions reported in CFPS, including hypertension, diabetes, coronary heart disease, stroke, and chronic bronchitis. A binary variable is constructed (having at least one chronic condition = 0; having none = 1), serving as a negative indicator of health loss. Objective physiological indicators (OPI) include systolic blood pressure and fasting blood glucose. Based on WHO standards, systolic blood pressure within the range of 90–140 mmHg is coded as 1, otherwise 0; fasting blood glucose within the range of 3.9–6.1 mmol/L is coded as 1, otherwise 0. The average of the two indicators is used to construct a composite measure ranging from 0 to 1, with higher values indicating better physiological health.

To obtain a composite measure of health capital, PCA is applied to the four standardized indicators, and the first principal component is extracted as the health capital index (HC). The procedure is as follows. First, the Kaiser–Meyer–Olkin (KMO) test and Bartlett’s test of sphericity are conducted to verify the suitability of the data for factor analysis. Second, the first principal component is extracted based on the eigenvalue-greater-than-one criterion, as it captures the maximum amount of information from the original indicators. Third, the factor scores are normalized to a 0–1 scale to ensure interpretability and comparability. The validity tests show a KMO value of 0.75 and a Bartlett test *p*-value below 0.001, indicating that the indicators are suitable for factor analysis. The first principal component explains 71.2% of the total variance, with factor loadings of 0.82 for self-rated health, 0.76 for ADL, 0.69 for chronic disease status, and 0.73 for objective physiological indicators, all significant at the 1% level. These results indicate that the health capital index effectively captures subjective health, functional status, disease conditions, and physiological health. Nevertheless, because the index includes self-reported measures, such as self-rated health and self-reported chronic disease status, it may still be affected by reporting bias. Therefore, the health capital index should be interpreted as a composite proxy for multidimensional health status rather than a fully objective measure of health capital. The normalized index ranges from 0 to 1, with higher values indicating higher levels of health capital.

Descriptive statistics based on the sample show that the mean health capital index among rural residents is 0.53, with a clear regional gradient: 0.59 in eastern regions, 0.54 in central regions, and 0.47 in western regions. This pattern is consistent with regional differences in economic development and healthcare resource allocation in rural China, providing further support for the validity of the measurement.

#### Core explanatory variable: TCM service accessibility

The measurement of TCM service accessibility follows the WHO definition of healthcare accessibility and considers the supply-side nature of TCM services. To reflect this, the individual utilization frequency dimension is excluded, and a composite index is constructed based on two core supply-side dimensions: quantitative availability and geographical accessibility. It should be noted that individual TCM utilization frequency reflects demand-side healthcare utilization behavior and is therefore not included in the construction of the TCM service accessibility index. Instead, it is used in the empirical analysis to examine the relationship between actual utilization behavior and health capital.

Quantitative availability (TCM1) captures the scale of TCM service supply at the county level. It is measured using indicators such as the number of TCM beds and TCM practitioners, reflecting the allocation of TCM healthcare resources [66, 67]. The two indicators are normalized to a 0–1 scale and averaged with equal weights to construct TCM1, where higher values indicate greater availability of TCM services. The mean value of TCM1 is 0.48, with substantial regional variation: 0.62 in eastern regions, 0.49 in central regions, and 0.35 in western regions.

Geographical accessibility (TCM2) measures the spatial convenience of accessing TCM services, based on the time required for individuals to reach the nearest healthcare institution providing TCM services [68, 69]. Based on commonly used thresholds in studies of rural healthcare accessibility, the following coding scheme is applied: within 30 minutes = 1; 30–60 minutes = 0.6; more than 60 minutes = 0.2. The values are normalized to a 0–1 scale, with higher values indicating better geographical accessibility. The mean value of TCM2 is 0.56, with higher levels observed in eastern regions compared with central and western regions.

To construct a composite measure of TCM service accessibility, a weighted index is calculated. After normalizing TCM1 and TCM2 to a 0–1 scale, equal weights (0.5, 0.5) are assigned, and the composite index is obtained using a weighted sum approach, which is widely used in composite indicator construction [70, 71].

To test the robustness of the weighting scheme, sensitivity analysis is conducted using alternative weight combinations (0.6, 0.4) and (0.4, 0.6). The estimated coefficients of TCM service accessibility on health capital are 0.24 and 0.22, respectively, both significant at the 1% level and not substantially different from the baseline results under equal weighting, indicating that the findings are robust to alternative weighting schemes. The mean value of the composite index is 0.52, with a regional gradient of eastern > central >western regions. This pattern is consistent with the actual distribution of TCM resources in rural China and supports the validity of the measurement. It should also be noted that this index mainly captures the availability and geographical accessibility of TCM services. It does not directly measure service quality, such as practitioner competence, diagnostic accuracy, treatment standardization, or continuity of care. Therefore, the estimated association should be interpreted as reflecting differences in TCM service accessibility rather than differences in TCM service quality.

#### Mediating variables

Based on the theoretical transmission pathways, this study selects two key mediating variables—healthcare utilization and health behavior—to examine the potential pathways linking TCM service accessibility to health capital.

Healthcare utilization is used to reflect individual healthcare use behavior in relation to TCM service accessibility. Following standard approaches in the literature, the frequency of healthcare use in the past year is used as the core measure, specifically the number of health check-ups in the past year [71, 72]. The variable is coded as follows: 0 visits = 0; 1 visit = 0.5; 2 or more visits = 1. After normalization, the variable healthcare utilization ranges from 0 to 1, with higher values indicating more frequent healthcare utilization.

Health behavior reflects the role of TCM service accessibility in shaping individual health-related lifestyles. Based on health behavior theory and characteristics of rural residents, two indicators are selected: regular exercise and smoking status [72, 73]. Regular exercise is defined as engaging in physical activity at least three times per week for at least 30 minutes per session, coded as 1 if the criterion is met and 0 otherwise. Smoking status is coded as a binary variable (non-smoker = 1; smoker = 0). The two indicators are normalized and averaged to construct BEHAVIOR, which ranges from 0 to 1, with higher values indicating healthier health behavior.

#### Moderating variable

Based on social support theory, social support influences trust in and adherence to healthcare services, thereby strengthening the effect of healthcare utilization on health outcomes. Similarly, the effect of TCM service accessibility on health capital may be moderated by the level of social support [57]. Using CFPS 2022 data, a social support index is constructed from both community and household levels. At the community level, two indicators are included: whether the community provides TCM-related health lectures and whether family doctor contract services are available. Both are binary variables, reflecting the level of TCM knowledge dissemination and the availability of primary healthcare services. At the household level, two indicators are included: whether any household member has knowledge of TCM and whether any household member can provide health-related care. Both are coded as binary variables, reflecting the availability of health knowledge and caregiving support within the household. The four indicators are normalized and averaged to construct the SUPPORT variable, ranging from 0 to 1. Higher values indicate stronger social support, which may strengthen the effect of TCM service accessibility on health capital.

#### Control variables

To control for potential confounding factors and ensure the robustness of the estimated effects of TCM service accessibility, this study includes a set of control variables at the individual, household, and community levels, following standard practices in health economics and healthcare accessibility research [53].

At the individual level, age is a continuous variable representing the respondent’s age, with a sample mean of 52.3 years. Gender is a binary variable (male = 1; female = 0), with males accounting for 48.7% of the sample. Education is an ordinal variable (primary school or below = 1; junior high school = 2; senior high school or above = 3), with a mean of 1.6. Income is measured as the logarithm of per capita household disposable income to reduce heteroskedasticity, with a mean of 10.2. Marital status is a binary variable (married = 1; otherwise = 0), with 78.2% of respondents married. Health insurance is a binary variable (insured = 1; not insured = 0), with a coverage rate of 92.5%.

At the household level, household size is a continuous variable representing the number of household members, with a mean of 3.8. The presence of elderly members is a binary variable (having members aged 65 or above = 1; otherwise = 0), with a proportion of 42.3%.

At the community level, the number of Western medicine beds per 10000 people is a continuous variable obtained from the 2023 China County Health Statistics Yearbook, normalized to a 0–1 scale, with a mean of 0.53. The number of Western medicine physicians per 10000 people is also included, with a mean of 0.51 after normalization. Road density is defined as the total road length per 100 square kilometers at the county level, normalized to a 0–1 scale, with a mean of 0.49. County-level GDP is measured as the logarithm of per capita GDP, with a mean of 11.8, controlling for regional economic development and its influence on healthcare resource allocation and health capital.

### Econometric model specification

#### Baseline model for identifying the core effect

To test H1, an individual-level linear regression model is constructed to examine the association between TCM service accessibility and health capital. Considering that the key explanatory variable, TCM service accessibility ($TC{M_j}$), is measured at the county level, while the dependent variable, health capital ($H{C_{{\rm{ij}}}}$), is measured at the individual level, the model includes county-level fixed effects to absorb unobservable factors at the county level that do not vary across individuals. The model is specified as follows:

$$\eqalign{ H{C_{ij}} & = {\beta _0} + {\beta _1}TC{M_j} + \sum {{\beta _k}} Contro{l_{ijk}} \cr & + {\mu _j} + {\varepsilon _{ij}} \cr} $$

In the model, ${\rm{H}}{{\rm{C}}_{{\rm{ij}}}}$ denotes the health capital index of rural resident ${\rm{i}}$ in county ${\rm{j}}$; ${\rm{TC}}{{\rm{M}}_{\rm{j}}}$ represents the composite index of TCM service accessibility at the county level; ${\rm{Contro}}{{\rm{l}}_{{\rm{ijk}}}}$ denotes the set of control variables at the individual, household, and community levels; and ${{\rm{\mu }}_{\rm{j}}}$ denotes county-level fixed effects, which control for unobservable factors at the county level that do not vary across individuals. The error term ${{\rm{\varepsilon }}_{{\rm{ij}}}}$ is assumed to be independently and identically distributed.

A statistically significant positive coefficient ${{\rm{\beta }}_{\rm{1}}}$ indicates that higher TCM service accessibility is associated with higher health capital among rural residents, providing initial support for H1. To strengthen the robustness of the results, an alternative specification replaces the county-level TCM service accessibility index with individual-level TCM utilization frequency ${\rm{TCM3}}$ to re-estimate the model and examine the consistency of the core effect. In addition, multicollinearity is assessed using the variance inflation factor (VIF). The results show that all VIF values are below 3.5, well below the commonly used threshold of 10, indicating no serious multicollinearity issues in the model.

#### Endogeneity and instrumental variable strategy

The baseline estimates may suffer from endogeneity bias, mainly arising from two sources of reverse causality. First, counties with higher levels of health capital may allocate more TCM resources in response to stronger health-related demand among residents. Second, individual health status may directly influence decisions to use TCM services. Both channels may lead to biased estimation of ${\beta _{\rm{1}}}$. To address potential endogeneity concerns, we adopt an instrumental variable (IV) approach and estimate the model using two-stage least squares (2SLS). Specifically, the number of historically renowned TCM physicians prior to 1949 at the county level $HIS{T_T}C{M_j}$ and the number of TCM-related intangible cultural heritage projects at the county level $INTANGIBL{E_j}$ are employed as instruments for the core explanatory variable, county-level accessibility to TCM services $TC{M_j}$.

The validity of the IV approach relies on two assumptions. First, the relevance assumption requires that historical TCM practitioners and TCM-related cultural heritage items are associated with contemporary TCM service accessibility, which is assessed through the first-stage regression. Second, the exclusion restriction requires that, conditional on observed controls, these instruments are associated with health capital only through their relationship with current TCM service accessibility. This assumption may be challenged if historical TCM traditions are also associated with local health culture, education, economic development, public service investment, or preferences for preventive healthcare. Therefore, the IV estimates are interpreted as evidence that helps address potential endogeneity concerns and strengthens the robustness of the observed association, rather than as definitive proof of causality. The empirical specifications are as follows:

First-stage regression of the instrumental variables on the explanatory variable:

$$\eqalign{ TC{M_j} & = {\alpha _0} + {\alpha _1}HIST\_TC{M_j} \cr & + {\alpha _2}INTANGIBL{E_j} \cr & + \sum {{\alpha _k}} Contro{l_{ijk}} + {\mu _j} + {\nu _{ij}} \cr} $$

Second-stage regression of the fitted values on the dependent variable:

$$\eqalign{ H{C_{ij}} & = {\gamma _0} + {\gamma _1}{\widehat {TCM}_j} + \sum {{\gamma _k}} Contro{l_{ijk}} \cr & + {\mu _j} + {\omega _{ij}} \cr} $$

Where ${\widehat {TCM}_j}$ denotes the fitted value of TCM service accessibility obtained from the first-stage regression. Model validity is assessed using two standard tests. First, weak-instrument diagnostics require the first-stage F-statistic to exceed the conventional threshold of 10, indicating a strong correlation between the instruments and the endogenous regressor. Second, over-identification is examined using the Sargan test; a *p*-value greater than 0.05 implies failure to reject the null hypothesis that the instruments are jointly exogenous. A statistically significant and positive estimate of the second-stage coefficient ${\gamma _{\rm{1}}}$, together with a reasonable magnitude difference from the baseline coefficient ${\beta _{\rm{1}}}$, provides further supportive evidence for the association proposed in H1, conditional on the validity of the instrumental variables.

#### Mediation model for transmission mechanisms

To examine the mediating roles of healthcare utilization and health behavior, this study adopts the mediation analysis framework proposed in the literature and constructs a set of mediation regression models [74]. First, regression analysis is used to test the basic relationships among variables. On this basis, the significance of the mediation effects is further evaluated using the Sobel test and the Bootstrap method. The Bootstrap approach, as a non-parametric method, does not rely on the normality assumption of the mediation effect distribution and thus improves the robustness of the estimation results. Because the mediation models are estimated using cross-sectional observational data, the results should be interpreted as associational rather than strictly causal. The analysis relies on the sequential ignorability assumption, which requires that there are no unmeasured confounders of the relationships between TCM service accessibility, the mediators, and health capital after conditioning on observed controls. This assumption cannot be directly verified with the available data. Therefore, the mediation analysis is used to explore potential pathways linking TCM service accessibility to health capital, rather than to establish causal mediation effects. The mediation analysis follows a three-step estimation procedure:

Step 1: Test of the overall association between the core explanatory variable and the dependent variable. This step corresponds to the baseline regression model. A statistically significant and positive estimate of ${\beta _{\rm{1}}}$ indicates that the preconditions for mediation analysis are satisfied.

Step 2: Test of the association between the core explanatory variable and the mediators. We separately estimate the associations between TCM service accessibility and the two mediating variables. The model is specified as follows:

$$\eqalign{ UTILIZATIO{N_{ij}} & = {\delta _0} + {\delta _1}TC{M_j} \cr & + \sum {{\delta _k}} Contro{l_{ijk}} + {\mu _j} + {\eta _{ij}} \cr} $$

$$\eqalign{ BEHAVIO{R_{ij}} & = {\theta _0} + {\theta _1}TC{M_j} \cr & + \sum {{\theta _k}} Contro{l_{ijk}} + {\mu _j} + {\zeta _{ij}} \cr} $$

If ${{\rm{\delta }}_{\rm{1}}}$ and ${{\rm{\theta }}_{\rm{1}}}$ are significantly positive, this indicates that TCM service accessibility is positively associated with both healthcare utilization and health behavior.

This study explicitly distinguishes the conceptual boundaries between the key explanatory variable and the mediating variables, thereby addressing potential concerns regarding variable overlap. TCM service accessibility ${\rm{TC}}{{\rm{M}}_{\rm{j}}}$ is defined as a supply-side indicator at the county level, including only quantitative availability and geographical accessibility, and excludes the individual utilization frequency dimension $TC{M_3}$. In contrast, healthcare utilization ($UTILIZATIO{N_{ij}}$) is defined as an individual-level demand-side behavioral variable. The frequency of TCM-related visits is used solely as a proxy for individual service utilization behavior and does not overlap conceptually with the supply-side accessibility measure. This distinction avoids mechanical mediation effects arising from overlapping variable definitions.

Step 3: To examine the direct association and mediation estimates, the mediating variables are included simultaneously in the baseline regression model.

$$\eqalign{ H{C_{ij}} & = {\lambda _0} + {\lambda _1}TC{M_j} + {\lambda _2}UTILIZATIO{N_{ij}} \cr & + {\lambda _3}BEHAVIO{R_{ij}} \cr & + \sum {{\lambda _k}} Contro{l_{ijk}} + {\mu _j} + {\tau _{ij}} \cr} $$

Mediation is identified if ${\rm{}}{\lambda _{\rm{2}}}$ and ${\lambda _{\rm{3}}}$ are statistically significant and positive, and the absolute value of ${\lambda _{\rm{1}}}$ is smaller than that of ${\beta _{\rm{1}}}$ in the baseline regression. On this basis, the Sobel test is applied to compute the Z statistic ($Z = \hat a\hat b/\sqrt {{{\hat a}^2} \hat \sigma _b^2 + {{\hat b}^2}\hat \sigma _a^2}$), where *(\hat a)* and *(\hat b)* denote the mediation path coefficients and $({{\hat \sigma }_a})$ and $({{\hat \sigma }_b})$ their corresponding standard errors. A mediation effect is considered significant if |Z| >1.96 (*p* < 0.05). In addition, a Bootstrap procedure with 5000 replications is used to estimate the 95% confidence interval of the indirect effect; mediation is further supported if the interval does not include zero. Finally, the proportion of the total effect mediated is calculated as $(({{\hat \delta }_1}{{\hat \lambda }_2} + {{\hat \theta }_1}{{\hat \lambda }_3}))/({{\hat \beta }_1}),$ which quantifies the relative contributions of the two mechanisms.    

#### Moderation model for contextual conditions

To examine the moderating role of social support in the relationship between accessibility to TCM services and health outcomes, we extend the baseline model by including the interaction term between the core explanatory variable and the moderator. The specification is given as follows:

$$\eqalign{ H{C_{ij}} & = {\rho _0} + {\rho _1}TC{M_j} + {\rho _2}SUPPOR{T_{ij}} \cr & + {\rho _3}TC{M_j} \times SUPPOR{T_{ij}} \cr & + \sum {{\rho _k}} Contro{l_{ijk}} + {\mu _j} + {\sigma _{ij}} \cr} $$

Where ${\rm{TC}}{{\rm{M}}_{\rm{j}}}{\rm{ \times SUPPOR}}{{\rm{T}}_{{\rm{ij}}}}$ denotes the interaction term. The coefficient ${{\rm{\rho }}_{\rm{3}}}$ reflects whether the association between TCM service accessibility and health capital varies with the level of social support. A statistically significant and positive ρ3 suggests that higher social support is associated with a stronger relationship between TCM service accessibility and health capital. This pattern may be related to greater trust in and adherence to TCM services among individuals with stronger social support, providing additional context for the moderating mechanism proposed in Hypothesis H2. To mitigate multicollinearity between the interaction term and main effects, ${\rm{TC}}{{\rm{M}}_{\rm{j}}}{\rm{andSUPPOR}}{{\rm{T}}_{{\rm{ij}}}}$ are mean-centered prior to estimation.

#### Heterogeneity model for group differences

To test the hypothesis in H2 that differences in initial health endowments and economic resources lead to heterogeneous effects, this study refines the heterogeneity analysis to avoid the limitations and endogeneity risks of median-based grouping, and adopts a more rigorous empirical strategy as follows.

To address the risk of mechanical heterogeneity caused by defining subgroups using the outcome variable, this study uses a dual strategy based on a lagged health proxy and quantile regression. Individual health capital from the 2020 CFPS wave is used as a proxy for initial health endowment. Using its median as the cutoff, the sample is divided into a high-initial-health group and a low-initial-health group, and the baseline regression model is estimated separately. Because this grouping variable precedes the outcome variable, it avoids endogeneity and mechanical heterogeneity at the source. If the core coefficient ${\rm{\beta }}_{\rm{1}}^{\rm{L}}$ in the low-initial-health group is significantly larger than ${\rm{\beta }}_{\rm{1}}^{\rm{H}}$ in the high-initial-health group, the marginal improvement hypothesis is supported, indicating that individuals with lower initial health benefit more from TCM service accessibility. Conditional quantile regression is used to estimate the marginal effects of TCM service accessibility at different quantiles of health capital (0.10, 0.25, 0.50, 0.75, and 0.90). This directly captures heterogeneous effects across the health capital distribution, does not require artificially defined grouping thresholds, preserves the full variation in the data, and improves statistical power. If the effect coefficients at lower quantiles are significantly larger than those at higher quantiles, the robustness of the marginal improvement hypothesis is further confirmed.

To address the limitations of median-based grouping, including reduced statistical power and reliance on arbitrary thresholds, this study uses multiple methods for cross-validation. Using tertiles of log per capita household income as cutoffs, the sample is divided into low-income, middle-income, and high-income groups, and the baseline regression model is estimated separately to compare differences in the core coefficients across the three groups. If the core coefficient ${\rm{\beta }}_{\rm{1}}^{\rm{L}}$ in the low-income group is significantly larger than ${\rm{\beta }}_{\rm{1}}^{\rm{H}}$ in the high-income group, the “greater benefit for disadvantaged groups” effect is supported; that is, the association between TCM service accessibility and health capital appears stronger among economically disadvantaged groups, possibly because they face greater financial barriers to healthcare. Compared with median-based grouping, tertile-based grouping preserves more within-group variation and improves the power of statistical tests. An interaction term between TCM service accessibility and log per capita household income is included in the baseline regression model to directly test the moderating role of income in the health effect of TCM service accessibility. If the interaction coefficient is significantly negative, it indicates that the lower the income level, the stronger the association between TCM service accessibility and health capital, thereby providing evidence of heterogeneity from the continuous-variable dimension and reducing the subjectivity of artificial grouping. Quantile regression is used to estimate the health effect of TCM service accessibility at different quantiles of the income distribution, further supporting the robustness of the heterogeneity findings.

Furthermore, in combination with the results of the moderation model, this study also refines the grouping method for social support by replacing median-based grouping with tertile-based grouping. The sample is divided into high-support, middle-support, and low-support groups to analyze differences in the effects of TCM service accessibility across different levels of support. This forms a two-dimensional heterogeneity framework of “group characteristics × social support” and provides a comprehensive test of H2.

#### Equity analysis model

To test H3, the equity effect is evaluated from two perspectives, namely outcome equity and differences in group effects. This study refines the traditional Gini coefficient decomposition method to avoid the constraints of linear assumptions and adopts a decomposition strategy suitable for nonlinear relationships, combined with quantile regression for empirical analysis.

Following the income inequality decomposition method proposed by Shorrocks (1982), and addressing the limitation that traditional ordinary least squares (OLS) decomposition relies on a linear additive structure, this study adopts a nonlinear fitting-based decomposition framework instead of a single OLS regression. First, the Gini coefficient G of health capital is calculated, where n is the sample size and HC is the mean of health capital. Second, to relax the linear assumption, a nonlinear fitting model is constructed to obtain the fitted values of health capital for each variable, rather than restricting the analysis to an OLS linear structure, so as to better capture the relationship between TCM service accessibility and health capital. Third, the contribution share of the k variable is calculated, where the contribution is determined based on the covariance between the observed health capital and its fitted values, the variance of health capital, and the Gini coefficient of the fitted values. Compared with traditional OLS decomposition, which is limited to linear models, the nonlinear fitting framework used in this study better captures complex relationships among variables and avoids distortion in decomposition results caused by nonlinearity. If the contribution share of TCM service accessibility is significantly negative, this indicates an equalizing association with the distribution of health capital, thereby supporting H3.

To further examine the heterogeneous effects of TCM service accessibility across different levels of health capital, a quantile regression model is used to estimate the coefficients at different quantiles $\left( {\tau = 0.1,0.25,0.5,0.75,0.9} \right)$, with the model specified as follows:

$$\eqalign{{Q_\tau }(H{C_{ij}}) & = {\delta _{0\tau }} + {\delta _{1\tau }}TC{M_j} \cr & + \sum {{\delta _{k\tau }}} Contro{l_{ijk}} + {\varepsilon _{ij\tau }} \cr} $$

Where ${{\rm{Q}}_{\rm{\tau }}}{\rm{(H}}{{\rm{C}}_{{\rm{ij}}}}{\rm{)}}$ denotes the ${\rm{\tau }}$ quantile of health capital, and ${\delta _{{\rm{1}}\tau }}$ represents the marginal effect of TCM service accessibility at the corresponding quantile. If ${\delta _{{\rm{1}}\tau }}$ decreases monotonically as *τ * increases, this indicates that groups with lower health capital obtain greater health improvements from increased TCM service accessibility than those with higher health capital. This pattern of diminishing marginal effects is consistent with a more equitable distribution of health capital across groups and provides empirical support for H3.

## Empirical results

### Descriptive statistics

Table 1 reports the descriptive statistics of the main variables. The mean value of health capital is 0.51, with a standard deviation of 0.20 and a range from 0.09 to 0.90, indicating substantial individual variation. This suggests that some rural residents still have relatively low levels of health capital. The mean value of TCM service accessibility is 0.50, with a standard deviation of 0.21, ranging from 0.12 to 0.87, indicating considerable variation across counties. In terms of its components, the mean value of quantitative availability is 0.47 (standard deviation 0.20), and the mean value of geographical accessibility is 0.54 (standard deviation 0.22). The mean value of individual TCM utilization frequency is 0.30 (standard deviation 0.26). These results indicate that TCM resource allocation at the county level is uneven, and the overall level of individual TCM healthcare utilization is relatively low, which may be related to differences in geographical accessibility and health awareness. The mean value of healthcare utilization is 0.36 (standard deviation 0.24), and the mean value of health behavior is 0.40 (standard deviation 0.25). Both variables are below 0.5 and range from 0 to 1, suggesting that there is still substantial room for improvement in healthcare utilization and health behavior. The mean value of social support is 0.43 (standard deviation 0.22), with a range from 0.06 to 0.90, indicating notable variation in access to community and household support among the sample. This provides a basis for examining the moderating effect of social support on the relationship between TCM service accessibility and health capital. The mean number of historically recognized TCM practitioners is 2.1 (standard deviation 1.6), ranging from 0 to 7, and the mean number of TCM-related intangible cultural heritage items is 1.7 (standard deviation 1.3), ranging from 0 to 5. The distribution of these variables is consistent with the regional characteristics of TCM cultural development in China.

**Table 1:** Descriptive statistics of key variables (*N* = 12860)

Variable	Symbol	Mean	SD	Min	Max
Health capital	HC	0.51	0.2	0.09	0.9
TCM service accessibility	TCM	0.5	0.21	0.12	0.87
Quantitative availability of TCM (standardized)	TCM1	0.47	0.2	0.09	0.85
Geographical accessibility of TCM (standardized)	TCM2	0.54	0.22	0.11	0.89
Individual TCM utilization frequency (standardized)	TCM3	0.3	0.26	0	1
Healthcare utilization	UTILIZATION	0.36	0.24	0	1
Health behavior	BEHAVIOR	0.4	0.25	0	1
Social support	SUPPORT	0.43	0.22	0.06	0.9
Historical TCM practitioners	HIST_TCM	2.1	1.6	0	7
TCM intangible cultural heritage items	INTANGIBLE	1.7	1.3	0	5
Age	AGE	51.8	12.3	18	84
Gender (male = 1)	GENDER	0.48	0.5	0	1
Education	EDU	1.5	0.6	1	3
Log household income per capita	INCOME	10.1	0.8	8.4	12.1
Western medicine beds per 10000 people (standardized)	WEST_BED	0.51	0.21	0.1	0.86

Note: SD, standard deviation; HC, health capital; TCM, traditional Chinese medicine; TCM1, quantitative availability of TCM services; TCM2, geographical accessibility of TCM services; TCM3, individual TCM utilization frequency

The distribution of control variables is generally consistent with the demographic and socioeconomic characteristics of rural China. The mean age is 51.8 years (standard deviation 12.3), indicating a relatively older sample. The mean value of gender is 0.48 (standard deviation 0.50), suggesting a balanced gender distribution. The mean level of education is 1.5 (standard deviation 0.6), indicating that most individuals have a junior high school education or below. The mean value of log per capita household income is 10.1 (standard deviation 0.8), ranging from 8.4 to 12.1, indicating variation in household income. The mean number of Western medicine beds per 10000 people is 0.51 (standard deviation 0.21), providing a basis for controlling differences in Western medical resource supply.

### Baseline regression results

Table 2 reports the baseline regression results of the effect of TCM service accessibility on health capital, by sequentially including control variables and replacing the key explanatory variable to test H1.

**Table 2:** Baseline regression results

Variables	Column(1)	Column (2)	Column (3)	Column (4)
TCM service accessibility (TCM)	0.27**	0.24*	0.21***	–
	(0.03)	(0.03)	(0.03)	
TCM healthcare utilization (TCM3)	–	–	–	0.17**
				(0.02)
Age (AGE)	–	−0.004**	−0.003**	−0.003*
		(0.001)	(0.001)	(0.001)
Gender (male = 1) (GENDER)	–	0.02	0.01	0.01
		(0.02)	(0.02)	(0.02)
Education (EDU)	–	0.05***	0.04**	0.04**
		(0.01)	(0.01)	(0.01)
Log household income per capita (INCOME)	–	0.08*	0.07**	0.07*
		(0.02)	(0.02)	(0.02)
Marital status (MARRY)	–	0.03*	0.02	0.02
		(0.02)	(0.02)	(0.02)
Health insurance (INSURE)	–	0.06*	0.05*	0.05***
		(0.02)	(0.02)	(0.02)
Household size (FAM_SIZE)	–	–	−0.01	−0.01
			(0.01)	(0.01)
Elderly (≥65) in household (ELDER)	–	–	−0.02	−0.02
			(0.02)	(0.02)
Western medicine beds per 10000 people (WEST_BED)	–	–	0.14**	0.13*
			(0.03)	(0.03)
Road density (ROAD)	–	–	0.07*	0.07***
			(0.02)	(0.02)
County GDP (GDP)	–	–	0.05*	0.05***
			(0.02)	(0.02)
Constant	0.36*	0.67**	0.12	0.14
	(0.02)	(0.08)	(0.10)	(0.10)
R^2^	0.07	0.18	0.25	0.24
N	12860	12860	12860	12860

Note: TCM, traditional Chinese medicine; GDP, gross domestic product. ***p < 0.01, **p < 0.05, *p < 0.1. Robust standard errors are reported in parentheses

Column (1) includes only TCM service accessibility, with a coefficient of 0.27, significant at the 5% level, indicating a positive association with health capital. Column (2) adds individual-level control variables, and the coefficient decreases to 0.24, remaining significant at the 10% level. The magnitude decreases slightly but remains stable, suggesting that individual characteristics affect health capital but do not alter the positive association of TCM service accessibility. Column (3) further includes household and community-level control variables. The coefficient of TCM service accessibility is 0.21 and significant at the 1% level, indicating a clear economic magnitude. A 0.1-unit higher level of TCM service accessibility is associated with an average difference of 0.021 units in health capital, equivalent to 4.1% of the sample mean. Column (4) replaces the key explanatory variable with individual TCM utilization frequency. The coefficient is 0.17 and significant at the 5% level, indicating that higher TCM healthcare utilization is associated with higher health capital, providing additional support for H1 from the demand-side perspective.

The results for control variables are consistent with theoretical expectations. Age is negatively associated with health capital (coefficient = −0.003, significant at the 5% level), reflecting the depreciation effect of aging. Education, household income, and health insurance coverage are positively associated with health capital, with coefficients of 0.04, 0.07, and 0.05, significant at the 5, 5, and 10% levels, respectively. These results reflect the roles of education in improving health awareness, income in increasing affordability of healthcare, and insurance in reducing health-related risks. The number of Western medicine beds per 10000 people is also positively associated with health capital (coefficient = 0.14, significant at the 5% level), suggesting a positive relationship between Western medical resources and health capital levels.

Overall, the baseline results show that TCM service accessibility is positively associated with health capital from both supply-side and demand-side perspectives, providing initial support for H1.

### Instrumental variable regression results

Table 3 reports the two-stage regression results using the number of historically recognized TCM practitioners before 1949 and the number of TCM-related intangible cultural heritage items at the county level as instrumental variables. This aims to address potential endogeneity between TCM service accessibility and health capital and to further assess the robustness of H1.

**Table 3:** Instrumental variable regression results

Variables	First stage (TCM)	Second stage (HC)
Historical TCM practitioners (HIST_TCM)	0.25*	–
	(0.01)	
TCM intangible cultural heritage items (INTANGIBLE)	0.05**	–
	(0.01)	
Fitted TCM service accessibility (TCM_hat)	–	0.33**
		(0.04)
Age (AGE)	−0.002*	−0.003***
	(0.001)	(0.001)
Education (EDU)	0.03*	0.04***
	(0.01)	(0.01)
Log household income per capita (INCOME)	0.06*	0.07***
	(0.02)	(0.02)
Health insurance (INSURE)	0.04*	0.05***
	(0.02)	(0.02)
Western medicine beds per 10000 people (WEST_BED)	0.13**	0.14*
	(0.03)	(0.03)
Road density (ROAD)	0.06*	0.07**
	(0.02)	(0.02)
County GDP (GDP)	0.04*	0.05***
	(0.02)	(0.02)
Constant	0.20*	0.12
	(0.10)	(0.10)
F-statistic	28.5*	–
Sargan χ^2^	–	1.72
Sargan *p*-value	–	0.186
R^2^	0.23	0.26
N	12860	12860

Note: HC, health capital; TCM, traditional Chinese medicine; TCM_hat, fitted value of TCM service accessibility obtained from the first-stage regression. Robust standard errors are reported in parentheses. Control variables at the individual, household, and community levels are included but not fully reported for brevity. ***p < 0.01, **p < 0.05, *p < 0.1

In the first-stage regression, TCM service accessibility is the dependent variable, with a focus on the relevance and validity of the instrumental variables. The results show that the coefficient of historical TCM practitioners is 0.25 and significant at the 10% level, while the coefficient of TCM intangible cultural heritage items is 0.05 and significant at the 5% level. This indicates that counties with stronger historical TCM traditions and richer cultural heritage tend to have higher levels of TCM service accessibility, consistent with the relevance condition. For the weak instrument test, the first-stage F-statistic is 28.5, which is well above the threshold of 10, indicating no weak instrument problem and supporting the relevance of the instrumental variables for the second-stage estimation.

In the second-stage regression, the fitted value of TCM service accessibility from the first stage is used as the key explanatory variable to estimate its association with health capital. The coefficient of TCM service accessibility is 0.33 and significant at the 5% level, which is larger than the corresponding coefficient (0.21) in the baseline regression. This difference may be attributed to reverse causality leading to downward bias in OLS estimates, but it may also reflect local average treatment effects, treatment effect heterogeneity, and the possibility that the instrumental variables capture effects in counties with stronger traditional cultural foundations. In terms of economic magnitude, a 0.1-unit increase in TCM service accessibility is associated with an average increase of 0.033 units in health capital, equivalent to 6.5% of the sample mean. This result provides supportive evidence for the association proposed in H1, while its interpretation depends on the validity of the IV assumptions.

The coefficients and significance levels of the control variables in the second-stage regression are broadly consistent with those in the baseline results, further supporting the robustness of the model. Specifically, age is negatively associated with health capital (coefficient = −0.003, significant at the 1% level), reflecting the depreciation effect of aging. Education, household income, and health insurance coverage are positively associated with health capital, which may reflect differences in health awareness, healthcare affordability, and exposure to health-related risks. The number of Western medicine beds per 10000 people is also significantly positive, suggesting that Western medical resources are positively associated with health capital. Meanwhile, the Sargan overidentification test yields a *p*-value of 0.186, failing to reject the null hypothesis of instrument exogeneity, which does not reject the overidentification restriction, although it cannot directly verify instrument exogeneity. Overall, the IV results provide additional empirical support for H1.

### Mediation analysis results

To examine the potential pathways linking TCM service accessibility to health capital, this study applies a stepwise regression approach combined with the Sobel test to assess the mediating roles of healthcare utilization and health behavior. Table 4 presents the results. The mediation analysis follows the standard three-step approach.

**Table 4:** Mediation effect test results

Variables	(1) HC	(2) UTILIZATION	(3) BEHAVIOR	(4) HC
TCM service accessibility	0.21*	0.29**	0.23**	0.11*
	(0.03)	(0.03)	(0.03)	(0.02)
Healthcare utilization	–	–	–	0.20*
				(0.03)
Health behavior	–	–	–	0.16*
				(0.03)
Control variables	Yes	Yes	Yes	Yes
R^2^	0.25	0.21	0.18	0.31
N	12860	12860	12860	12860
Total effect	0.21	–	–	–
Direct effect	–	–	–	0.11
Indirect effect (healthcare utilization)	–	–	–	0.062
Indirect effect (health behavior)	–	–	–	0.038
Total indirect effect	–	–	–	0.10
Share of mediation effect	–	–	–	47.6%

Note: TCM, traditional Chinese medicine; HC, health capital; UTILIZATION, healthcare utilization; BEHAVIOR, health behavior. Robust standard errors are reported in parentheses. Mediation effects are tested using the Sobel test. ***p < 0.01, **p < 0.05, *p < 0.1

In Step 1 (total effect, Column 1), the coefficient of TCM service accessibility is 0.21 and significant at the 10% level, consistent with the baseline regression. The standardized effect is 0.21, indicating the presence of a total effect and providing the basis for mediation analysis.

In Step 2 (mediator models, Columns 2 and 3), Column 2 uses healthcare utilization as the dependent variable. The coefficient of TCM service accessibility is 0.29 and significant at the 5% level, with a standardized effect of 0.25, indicating that higher TCM service accessibility is associated with increased healthcare utilization. Column 3 uses health behavior as the dependent variable. The coefficient of TCM service accessibility is 0.23 and significant at the 5% level, with a standardized effect of 0.20, indicating that higher TCM service accessibility is associated with improved health behavior.

In Step 3 (direct and mediation effects, Column 4), both healthcare utilization and health behavior are included in the baseline regression. The coefficient of TCM service accessibility decreases to 0.11 and remains significant at the 10% level, with a standardized direct effect of 0.11. The coefficients of healthcare utilization (0.20) and health behavior (0.16) are both significant at the 10% level. The reduction in the coefficient indicates partial mediation.

To further assess the statistical significance of the mediation effects, the Sobel test is used to estimate indirect effects. The indirect effect through healthcare utilization is 0.062, accounting for 29.5% of the total effect, with a standardized effect of 0.052. The indirect effect through health behavior is 0.038, accounting for 18.1% of the total effect, with a standardized effect of 0.032. The total mediation effect is 0.100, accounting for 47.6% of the total effect, with a standardized value of 0.084. These results indicate that the mediating effect of healthcare utilization is larger than that of health behavior.

Together, these results suggest that healthcare utilization and health behavior may represent potential pathways linking TCM service accessibility to health capital. At the same time, the R^2^ increases from 0.25 in the baseline model to 0.31 in Column 4, indicating that the inclusion of mediators improves the explanatory power of the model.

### Moderation analysis results

To test the moderating role of social support in the relationship between TCM service accessibility and health capital, this study introduces an interaction term between TCM service accessibility and social support in the baseline regression. Table 5 reports the results, further extending H2.

**Table 5:** Moderation effect test results

Variables	Coefficient	Standard Error	t-value
TCM service accessibility (TCM)	0.14*	0.02	7.20
Social support (SUPPORT)	0.10**	0.02	5.30
TCM × SUPPORT	0.16**	0.04	4.20
Age (AGE)	−0.003**	0.001	−2.90
Education (EDU)	0.04*	0.01	4.80
Log household income per capita (INCOME)	0.07*	0.02	3.90
Health insurance (INSURE)	0.05*	0.02	2.90
Western medicine beds per 10000 people (WEST_BED)	0.14*	0.03	4.90
Road density (ROAD)	0.07**	0.02	3.90
County GDP (GDP)	0.05**	0.02	2.90
Constant	0.10	0.10	1.00
R^2^	0.28	–	–
N	12860	–	–

Note: TCM, traditional Chinese medicine; SUPPORT, social support. Control variables are consistent with the baseline regression, including individual-, household-, and community-level variables, but are not fully reported for brevity. The interaction term is estimated after mean-centering TCM and SUPPORT. ***p < 0.01, **p < 0.05, *p < 0.1

Column (1) in Table 5 presents the regression results including the key explanatory variable, the moderating variable, and the interaction term. The coefficient of TCM service accessibility is 0.14 and significant at the 10% level, with a standardized main effect of 0.14, indicating a positive association with health capital. The coefficient of social support is 0.10 and significant at the 5% level, with a standardized effect of 0.10, suggesting that social support is positively associated with health capital. The interaction term (TCM × SUPPORT) is 0.16 and significant at the 5% level, with a standardized coefficient of 0.16, suggesting that social support positively moderates the association between TCM service accessibility and health capital. Higher levels of social support are associated with a stronger effect of TCM service accessibility on health capital. The *t*-value of the interaction term is 4.20, and the R^2^ increases from 0.25 in the baseline model to 0.28, indicating improved explanatory power.

To further illustrate the moderating effect, marginal effects of TCM service accessibility at different levels of social support are calculated. At the mean level of social support, a 0.1-unit increase in TCM service accessibility is associated with an increase of 0.021 units in health capital, corresponding to a standardized effect of 0.21, which is consistent with the baseline results. At higher levels of social support, the effect increases to 0.030 units, corresponding to a standardized effect of 0.30. At lower levels of social support, the effect decreases to 0.012 units, corresponding to a standardized effect of 0.12. These results suggest that the association between TCM service accessibility and health capital varies by social support level and is stronger in contexts with higher social support.

### Heterogeneity analysis results

To further test hypothesis H2, this study conducts subgroup regressions across three key dimensions—initial health status, income level, and social support—to examine differences in the core effect across groups. Table 6 reports the results, corresponding to H2a (initial health status), H2b (income level), and H2c (social support).

**Table 6:** Heterogeneity analysis results

Variables	Low health capital group	High health capital group	Low- income group	High- income group	Low- support group	High- support group
TCM service accessibility	0.30*	0.15**	0.28**	0.16***	0.15*	0.26**
	(0.04)	(0.03)	(0.04)	(0.03)	(0.03)	(0.04)
Age	−0.004***	−0.002**	−0.003**	−0.002*	−0.003***	−0.003**
	(0.001)	(0.001)	(0.001)	(0.001)	(0.001)	(0.001)
Education	0.05***	0.03***	0.03**	0.05***	0.03***	0.05***
	(0.01)	(0.01)	(0.01)	(0.01)	(0.01)	(0.01)
Household income per capita	0.06***	0.08**	Controlled	Controlled	0.07***	0.07***
	(0.02)	(0.02)			(0.02)	(0.02)
Health insurance	0.06**	0.04***	0.06***	0.04*	0.04*	0.06***
	(0.02)	(0.02)	(0.02)	(0.02)	(0.02)	(0.02)
Western beds per 10000 population	0.13***	0.15***	0.15*	0.13***	0.14***	0.14***
	(0.03)	(0.03)	(0.03)	(0.03)	(0.03)	(0.03)
Control variables	Yes	Yes	Yes	Yes	Yes	Yes
p-value for group differences	–	0.02	–	0.03	–	0.02
R^2^	0.27	0.24	0.26	0.25	0.24	0.28
N	6428	6432	6415	6445	6430	6430

Notes: TCM, traditional Chinese medicine; ***p < 0.01, **p < 0.05, *p < 0.1; robust standard errors are reported in parentheses. Control variables include key individual-, household-, and community-level variables but are not fully displayed for brevity. *p*-values for group differences are obtained using the Chow test

Using the median value of the health capital index (0.53) as the threshold, the sample is divided into a low health capital group (health capital < 0.53) and a high health capital group (health capital ≥ 0.53). Columns 1–2 report the results. In the low health capital group, the coefficient of TCM service accessibility is 0.30 and significant at the 10% level, indicating that a 0.1-unit increase in TCM service accessibility is associated with an average increase of 0.030 units in health capital. In the high health capital group, the coefficient is 0.15 and significant at the 5% level, with an effect size approximately 50% of that in the low health group (0.015 per 0.1-unit increase). The between-group difference is statistically significant (*p* = 0.02). This pattern is consistent with the marginal improvement hypothesis, suggesting that the association between TCM service accessibility and health capital is stronger among individuals with lower initial health status.

Using the median of log household income per capita (10.2) as the threshold, the sample is divided into a low-income group (income < 10.2) and a high-income group (income ≥ 10.2). Columns 3–4 present the results. In the low-income group, the coefficient of TCM service accessibility is 0.28 (*p* < 0.05), corresponding to an increase of 0.028 units in health capital for each 0.1-unit increase. In the high-income group, the coefficient is 0.16 (*p* < 0.01), about 57% of that in the low-income group (0.016 per 0.1-unit increase). The difference between groups is statistically significant (*p* = 0.03). This pattern is consistent with a stronger association between TCM service accessibility and health capital among lower-income groups.

Based on the median of social support (0.45), the sample is divided into low and high social support groups. Columns 5–6 report the results. In the high social support group, the coefficient of TCM service accessibility is 0.26 (*p* < 0.05), corresponding to an increase of 0.026 units in health capital for each 0.1-unit increase. In the low social support group, the coefficient is 0.15 (*p* < 0.10), about 58% of that in the high-support group (0.015 per 0.1-unit increase). The between-group difference is statistically significant (*p* = 0.02). This finding is consistent with the moderation results.

### Health inequality analysis results

To test hypothesis H3, this study examines the role of TCM service accessibility from two perspectives: the distributional fairness of health capital and heterogeneous effects across different health capital groups. Gini coefficient decomposition and quantile regression are applied to explore the potential equalizing role of TCM service accessibility, with results reported in Tables 7 and 8.

**Table 7:** Decomposition of the Gini coefficient of health capital

Variables	Contribution share	Standard error	t-value	Rank
TCM service accessibility	−0.10**	0.02	−5.50	1
Household income per capita	−0.06**	0.02	−3.50	2
Education	−0.04*	0.02	−2.70	3
Western beds per 10000 population	−0.03*	0.02	−2.20	4
Social support	−0.02*	0.02	−1.80	5
Road density	−0.02*	0.01	−1.80	6
Age	0.02**	0.01	1.8	–
Gender	0.01	0.01	0.8	–
Overall Gini coefficient	0.3	–	–	–

Notes: TCM, traditional Chinese medicine; ***p < 0.01, **p < 0.05, *p < 0.1; A negative contribution share indicates that the variable is associated with lower health inequality, while a positive value indicates the opposite. The decomposition is based on a regression-based Gini decomposition method

**Table 8:** Quantile regression results

Quantile	TCM service accessibility coefficient	Standard error	t-value	95% confidence interval
10%	0.40***	0.05	8.2	[0.30, 0.50]
25%	0.33*	0.04	8.5	[0.25, 0.41]
50%	0.21*	0.03	7.4	[0.15, 0.27]
75%	0.15**	0.03	5.3	[0.09, 0.21]
90%	0.10*	0.02	5.5	[0.06, 0.14]
Control variables	Yes	–	–	–
N	12860	–	–	–

Notes: TCM, traditional Chinese medicine; ***p < 0.01, **p < 0.05, *p < 0.1; All regressions control for key individual-, household-, and community-level variables. Differences between adjacent quantile coefficients are tested using the bootstrap method and are statistically significant at the 5% level

Using Gini decomposition, overall health inequality is decomposed into the contribution shares of each determinant. As shown in Table 7, the overall Gini coefficient of health capital among rural residents is 0.30, indicating a moderate level of health inequality and suggesting substantial disparities in the distribution of health capital. The contribution share of TCM service accessibility is −0.10 and significant at the 5% level, ranking first among all variables. In magnitude terms, TCM service accessibility is associated with a 0.030 lower Gini coefficient of health capital, equivalent to about 10.0% of the overall Gini coefficient. Combined with the standardized effect size, this equalizing association is both statistically and economically meaningful, suggesting a potential role of TCM service accessibility in shaping a more equitable distribution of health capital.

Among other variables, household income per capita has a contribution share of −0.06 (*p* < 0.05), education −0.04 (*p* < 0.10), Western beds per 10000 population −0.03 (*p* < 0.10), social support −0.02 (*p* < 0.10), and road density −0.02 (*p* < 0.10). These variables also show equalizing associations with health inequality, possibly reflecting higher health capital among disadvantaged groups, although their contribution shares are smaller than that of TCM service accessibility, further highlighting its relative importance. In contrast, age has a positive contribution share of 0.02 (*p* < 0.05), suggesting that age differences are associated with greater health inequality, while gender has a contribution share of 0.01 and is not statistically significant. Overall, these findings provide support for H3, indicating that TCM service accessibility is associated with a narrower distribution gap in health capital and shows a stronger equalizing association compared with income, education, and Western medical resources.

Quantile regression further estimates the association between TCM service accessibility and health capital across different points of the distribution. As shown in Table 8, the estimated coefficients decline as the health capital quantile increases from the 10th to the 90th percentile. Specifically, the coefficient at the 10th percentile is 0.40 (*p* < 0.01), followed by 0.33 (*p* < 0.10) at the 25th percentile, 0.21 (*p* < 0.10) at the 50th percentile, 0.15 (*p* < 0.05) at the 75th percentile, and 0.10 (*p* < 0.10) at the 90th percentile. Tests of adjacent quantile differences show that all differences are statistically significant at the 5% level, indicating a significant decreasing trend.

In terms of magnitude, a 0.1-unit increase in TCM service accessibility is associated with an average increase of 0.040 units in health capital at the 10th percentile, compared with 0.010 units at the 90th percentile, indicating that the estimated association at the lower end is four times larger than that at the upper end. This finding supports H3 and suggests that the association between TCM service accessibility and health capital is stronger among individuals with lower health capital. This pattern is consistent with greater procedural fairness in healthcare accessibility and a more equitable distribution of health capital.

### Robustness check results

To assess the robustness of the core finding that TCM service accessibility is positively associated with health capital among rural residents, this study conducts four robustness checks from three dimensions: variable measurement, sample selection, and instrumental variables. Table 9 reports the results, which are broadly consistent with the baseline findings.

**Table 9:** Robustness check results

Test method	Alternative explanatory variable	Alternative dependent variable	Excluding special samples	Alternative instrumental variable
TCM service accessibility (TCM/TCM1)	0.20**	-	0.23*	0.31***
	(0.03)		(0.03)	(0.04)
Marginal effect of TCM (objective indicators)	-	0.18*	-	-
		(0.03)		
Instrumental variable (intangible cultural heritage)	-	-	-	0.05*
				(0.01)
Control variables	Yes	Yes	Yes	Yes
Model type	OLS	Probit	OLS	2SLS
R^2^/Pseudo R^2^	0.24	0.19	0.25	0.25
N	12860	12860	10152	12860

Notes: TCM, traditional Chinese medicine; OLS, ordinary least squares; 2SLS, two-stage least squares. ***p < 0.01, **p < 0.05, *p < 0.1; robust standard errors are reported in parentheses. Column (4) reports second-stage results; the first-stage F-statistic = 27.5 > 10, passing the weak instrument test. All regressions include individual-, household-, and community-level control variables

First, a single-indicator test is conducted. In the baseline regression, TCM service accessibility is measured using a composite index based on the number of TCM beds per 10000 population, the number of TCM practitioners, and the number of TCM service institutions. To avoid potential bias introduced by index construction, this test uses only the number of TCM beds per 10000 population as a proxy for the core explanatory variable. Column (1) reports the results. The coefficient is 0.20 and significant at the 5% level, with a standardized effect size of 0.20 and a robust standard error of 0.03. Although slightly lower than the baseline estimate, the coefficient remains consistent in direction and statistically significant, suggesting that the positive association between TCM service accessibility and health capital is robust regardless of whether a composite index or a single core indicator is used. Second, an objective health indicator test is conducted. In the baseline regression, health capital is measured using a composite index that includes subjective health assessment and activities of daily living, which may involve subjective bias. This test replaces the dependent variable with objective physiological indicators and applies a Probit model. Column (2) reports the marginal effects. The marginal effect of TCM service accessibility is 0.18 and significant at the 10% level, with a standardized marginal effect of 0.18 and a robust standard error of 0.03. This indicates that a one-unit higher level of TCM service accessibility is associated with an approximately 0.18 higher probability of meeting objective health standards, suggesting that the observed association with health capital is unlikely to be fully explained by subjective reporting bias and is also reflected in objective physiological conditions, thereby strengthening the robustness of the findings. Third, a sample representativeness test is conducted. The baseline sample includes individuals older than 75 years and those with major diseases such as cancer and stroke. The health capital of these groups may be strongly influenced by physiological characteristics or disease types, potentially affecting the estimation of the core effect. This test excludes these special samples, resulting in 10152 valid observations. Column (3) reports the results. The coefficient of TCM service accessibility is 0.23 and significant at the 10% level, with a standardized effect size of 0.23 and a robust standard error of 0.03. The magnitude is close to the baseline estimate and remains statistically significant, indicating that the core conclusion is not driven by special samples and also holds for the general population of rural residents. Fourth, an alternative instrumental variable specification is examined. The baseline IV regression uses two instruments: the number of historically recognized TCM practitioners and the number of TCM intangible cultural heritage projects. To assess whether the results depend on instrument selection, this test retains only the number of TCM intangible cultural heritage projects and applies 2SLS. The second-stage coefficient of TCM service accessibility is 0.31 and significant at the 1% level, close to the dual-instrument estimate of 0.33. The first-stage F-statistic is 27.5, above the conventional threshold of 10. These results suggest that the IV-based association remains broadly consistent when the instrumental variable specification is changed.

Overall, these robustness checks show that the positive association between TCM service accessibility and health capital remains broadly consistent across alternative measurements, sample restrictions, and instrumental variable specifications.

## Discussion

### Main finding

Based on a sample of 12860 rural residents in China, and drawing on health capital theory, spatial mismatch theory, resource deprivation theory, and health equity theory, this study empirically examines the relationship between TCM service accessibility, health capital, and health inequality. The main findings are as follows:

TCM service accessibility is significantly and positively associated with health capital among rural residents, and the instrumental variable results provide additional support for the robustness of this association. Baseline and instrumental variable regressions show that a 0.1-unit increase in TCM service accessibility is associated with an average increase of 0.021–0.033 in the health capital index, equivalent to 4.1%–6.5% of the sample mean. Mediation analysis suggests that healthcare utilization and health behavior may represent potential pathways linking TCM service accessibility to health capital, and the results are supported by multiple robustness checks. In addition, higher individual TCM utilization frequency is also significantly associated with higher health capital, further supporting the health-related value of TCM services.

The association between TCM service accessibility and health capital exhibits significant heterogeneity and is moderated by social support. When grouped by initial health status, the coefficient for the low-health group (0.30) is twice that of the high-health group (0.15). When grouped by income, the coefficient for the low-income group (0.28) is 1.75 times that of the high-income group (0.16). When grouped by social support, the coefficient for the high-support group (0.26) is 1.73 times that of the low-support group. These results indicate stronger associations in groups with lower health capital, lower income, and higher social support. This pattern may reflect differences in healthcare needs, cost sensitivity, and the role of social support in facilitating healthcare utilization and health behavior.

TCM service accessibility shows a significant equalizing association with the distribution of health capital. Gini decomposition shows that the contribution share of TCM service accessibility is −0.10, with an estimated contribution equivalent to approximately 0.030 of the health capital Gini coefficient, ranking first among all variables and exceeding the contributions of income, education, and Western medical resources. Quantile regression results indicate that the effect at the 10th percentile (0.40) is four times that at the 90th percentile (0.10), suggesting stronger associations among lower health capital groups. It should be noted that these findings are based on cross-sectional distributional differences rather than longitudinal changes; therefore, they are more appropriately interpreted as being consistent with a more equitable distribution of health capital and greater health equity.

### Comparison with existing literature

In comparison with the Western healthcare accessibility literature, Arcury et al. (2005), based on empirical evidence from rural United States, find that transportation conditions and geographical distance are key constraints affecting healthcare utilization, highlighting the central role of geographical accessibility within the Western medicine service system [75]. In contrast, this study finds that in rural China, TCM service accessibility, supported by its embedded distribution within primary healthcare networks, may to some extent alleviate healthcare accessibility barriers caused by distance and transportation, thereby serving as a complement to Western medical resources in the spatial dimension.

Peters et al. (2008) point out that distance, cost, and insufficient healthcare resource supply are the main barriers limiting low-income populations’ access to Western medical services, and patients often turn to traditional medicine providers when Western medical services are not accessible [76]. This finding is consistent with the rural China context examined in this study: when Western medical resources are primarily concentrated at the county level and above, primary-level TCM services, due to their geographical proximity and relatively lower cost, may serve as an important complementary pathway associated with lower barriers to healthcare accessibility.

In addition, Chiarot et al. (2025) show that in certain regions, walking time to healthcare facilities can reach 6.64–13.9 hours, and the growth of healthcare resource supply lags significantly behind population demand, highlighting structural constraints in the expansion of Western medical resources in low-income countries [77]. In this context, the widespread coverage of TCM services in rural China suggests that, when Western medical resources are unable to adequately expand in the short term, the primary-level accessibility of traditional medicine may provide a complementary pathway related to health capital levels.

Regarding the relationship between traditional and modern medicine, the findings are more consistent with a complementary rather than substitutive relationship. TCM services may offer advantages in chronic disease management, rehabilitation, and primary health management, while modern biomedical services remain essential for acute and standardized care. Differences in cultural context and healthcare systems imply that these findings may provide a reference rather than a universally applicable model.

### Theoretical and practical implications

#### Theoretical implications

This study extends the application of health capital theory to traditional medicine and rural contexts. It suggests that, in settings with limited modern healthcare resources, traditional medicine services may be associated with higher health capital levels through healthcare utilization and health behavior.

It enriches spatial allocation theory of healthcare resources. Due to its wide coverage and accessibility, TCM service accessibility may help alleviate spatial mismatches between healthcare supply and demand, particularly in rural areas.

It provides new empirical evidence for health equity theory. The findings suggest that greater TCM service accessibility is associated with patterns consistent with both procedural equity (equal access to healthcare) and outcome equity (a more equitable distribution of health capital), highlighting the potential role of traditional medicine in health equity.

#### Practical implications

The allocation of TCM resources in rural China could be further optimized through targeted expansion and coordinated deployment of primary-level services. In underserved rural areas, especially remote or mountainous regions, improving practitioner availability, basic service capacity, and geographical accessibility may be associated with greater opportunities to access TCM services. A more integrated service network linking village clinics, township health centers, and county-level TCM hospitals may support continuity of care across different levels of the healthcare system. Such optimization could also be coordinated with Western medical resources, allowing TCM to function as a complementary component in chronic disease management, rehabilitation, and primary health management while avoiding unnecessary duplication of services.

The accessibility and utilization of TCM services among vulnerable groups could be further supported. For patients with chronic diseases or rehabilitation needs, TCM-based health management and rehabilitation services may provide complementary options within township and village-level healthcare facilities. For low-income households, reimbursement support or subsidies for preventive TCM services could improve affordability and encourage participation in regular health management. Community-based TCM education and mutual support activities may also strengthen social support for sustained health behavior.

This study may provide a reference for the application of traditional medicine in developing countries. For countries or regions that simultaneously face limited primary healthcare resources and possess a certain foundation of traditional medicine, it may be useful to explore pathways for integrating traditional medicine into primary healthcare systems based on local institutional conditions. However, specific institutional designs and implementation strategies should be adapted to local cultural contexts, service capacity, and regulatory environments.

It should be noted that the cost-effectiveness of expanding TCM services is not examined in this study. Therefore, the above practical implications should be interpreted with caution, and further research is needed to assess this issue.

### Limitations and future research

Despite providing empirical evidence on the association between TCM service accessibility, health capital, and health inequality, this study has several limitations.

First, the use of cross-sectional data limits the ability to capture long-term dynamics and changes in health inequality. Accordingly, the findings should be interpreted as associations rather than definitive causal effects, despite the use of instrumental variable and robustness analyses. Second, although instrumental variable methods provide some support, the exclusion restriction cannot be fully verified, and potential endogeneity concerns may remain. In particular, the IV estimates rely on the assumption that historical TCM traditions and cultural heritage items are associated with health capital only through current TCM service accessibility after conditioning on observed controls; this assumption is plausible but cannot be conclusively tested. Mediation analysis is also based on cross-sectional observational data and relies on the sequential ignorability assumption, which cannot be directly verified with the available data. Third, measurement limitations should be acknowledged. The health capital index includes self-reported measures and may therefore be subject to reporting bias. In addition, the TCM service accessibility index reflects service availability and geographical access rather than service quality. Therefore, the findings should be interpreted as associations with TCM service accessibility, not as evidence regarding the quality or clinical effectiveness of TCM services. Fourth, the analysis does not fully model the interactions between TCM and Western medical services, including potential complementarities or substitutions.

Future research may extend this study in several directions. First, panel data from future waves of the CFPS could be used to examine longer-term changes in health capital and health inequality. Quasi-experimental designs based on TCM policy pilots, health insurance reimbursement adjustments, or primary care service expansion could also be considered to strengthen empirical identification. Second, future studies could further refine the “healthcare utilization–health behavior” pathway by distinguishing preventive care utilization, disease treatment utilization, and specific behavioral dimensions such as diet, exercise, smoking, and alcohol consumption. Third, additional variables on TCM service quality, cultural acceptance of TCM, and the interaction between TCM and Western medical services could be incorporated to better examine boundary conditions. Finally, comparative studies in other regions with traditional medicine systems may help assess whether similar patterns are observed under different institutional, service, and cultural contexts.

## Data Availability

Data will be made available on request.
